# Secular Trends in Depressive Symptoms in Adolescents in Yunnan, Southwest China From Before COVID-19 to During the COVID-19 Pandemic: Longitudinal, Observational Study

**DOI:** 10.2196/52683

**Published:** 2024-07-31

**Authors:** Yunjuan Yang, Shun Zha, Tunan Li

**Affiliations:** 1 Public Health School Xi'an Jiaotong University Xi China; 2 Department of School Health Yunnan Provincial Center for Disease Control and Prevention Kunming China; 3 Public Health School Kunming Medical University Kunming China; 4 Public Health School Dali University Dali China

**Keywords:** COVID-19 exposure, depressive symptom, adolescent, epidemic trend, prevalence, observational study, epidemic, COVID-19, depression, symptoms, teen, youth, China, mental health, psychological, logistic regression, lifestyle intervention

## Abstract

**Background:**

Yunnan province borders Myanmar, Laos, and Vietnam, giving it one of the longest borders in China. We aimed to determine the trends in prevalence and impact of COVID-19 on depressive symptoms among adolescents (12-18 years) from 2018 to 2022 in Yunnan, southwest China.

**Objective:**

We evaluated the impact of the COVID-19 epidemic on adolescents’ mental health, with the aim of reducing the effect of psychological emergency syndrome and promoting healthy, happy adolescent growth.

**Methods:**

This longitudinal, observational study used Students’ Health Survey data on adolescents’ depressive symptoms from 2018 to 2022 (before and during COVID-19) in Yunnan. We used multistage, stratified sampling in 3 prefectures in 2018 and 16 prefectures from 2019 to 2022. In each prefecture, the study population was classified by gender and residence (urban or rural), and each group was of equal size. Depressive symptoms were diagnosed based on Center for Epidemiological Studies Depression Scale (CES-D) scores. We used ANOVA to assess the differences in mean CES-D scores stratified by gender, age, residence, grade, and ethnicity. Chi-square tests were used to compare depressive symptoms by different variables. For comparability, the age-standard and gender-standard population prevalences were calculated using the 2010 China Census as the standard population. The association between COVID-19 and the risk of a standardized prevalence of depressive symptoms was identified using unconditional logistic regression analysis.

**Results:**

The standardized prevalence of depressive symptoms for all participants was 32.98%: 28.26% in 2018, 30.89% in 2019, 29.81% in 2020, 28.77% in 2021, 36.33% in 2022. The prevalences were 30.49% before COVID-19,29.29% in early COVID-19, and 36.33% during the COVID-19 pandemic. Compared with before COVID-19, the risks of depressive symptoms were 0.793 (95% CI 0.772-0.814) times higher in early COVID-19 and 1.071 (95% CI 1.042-1.100) times higher than during COVID-19. The average annual increase in depressive symptoms was 1.61%. During the epidemic, the prevalence of depressive symptoms in girls (36.87%) was higher than that in boys (28.64%), and the acceleration rate of girls was faster than that of boys. The prevalences of depressive symptoms and acceleration rates by age group were as follows: 27.14% and 1.09% (12-13 years), 33.99% and 1.8% (14-15 years), 36.59% and 1.65% (16-18 years). Prevalences did not differ between Han (32.89%) and minority (33.10%) populations. However, the acceleration rate was faster for the former than for the latter. The rate for senior high school students was the highest (34.94%). However, the acceleration rate for vocational high school students was the fastest (2.88%), followed by that for junior high school students (2.32%). Rural residents (35.10%) had a higher prevalence and faster acceleration than urban residents (30.16%).

**Conclusions:**

From 2018 to 2022, there was a significant, continuous increase in the prevalence of depressive symptoms among adolescents in Yunnan, China, especially during the COVID-19 pandemic. This represents an emergency public health problem that should be given more attention. Effective, comprehensive psychological and lifestyle intervention measures should be used to reduce the prevalence of mental health issues in adolescents.

## Introduction

Adolescence is a critical period for brain, cognitive, and emotional development as well as individual growth and development. It is also a high-risk period for the development of psychological problems and serious internalization problems, including anxiety and depression [1,2].

With the COVID-19 pandemic, which resulted in a rapidly changing world with major social changes, demands, and lifestyle changes, multiple crises exposed adolescents to increasing stressors that affected their mental health [3,4]. In particular, anxiety and depressive disorders increased [5,6]. Adolescents face a great threat of anxiety and depressive disorders [7]. A study in Oman showed that 13.9% of children and adolescents exhibited depressive symptoms during the COVID-19 pandemic [8], and other studies also reported that the proportion of adolescents who reported suicidal ideation and self-harm increased significantly [9,10]. By 2030, depressive disorders are predicted to become the largest burden of disease in the world and result in major public health challenges [11].

Depressive disorders in adolescents have a negative impact on sleep, psychosocial functioning, and academic performance. It is an important predictive factor of suicide [12]. It may have serious impacts on adolescents' overall well-being and functioning, with long-term consequences into adulthood [13,14]. With the increasing clinical prevalence and rate of adolescent depressive disorders, adolescent depressive symptoms have attracted a high level of concern as a precursor for depressive disorders in the clinical stage.

According to a meta-analysis, 1 in 4 youths globally experienced clinical depressive symptoms during the first year of the COVID-19 pandemic, and this number doubled after the pandemic [15,16]. In China, more than 50% of respondents in a study experienced psychological effects during the beginning of the COVID-19 pandemic [17]. Many foreign researchers have concluded that most people experienced various degrees of depressive symptoms with the increasing waves of the COVID-19 pandemic [18-21]. Due to the rare convergence of public health crises, the COVID-19 pandemic may have exacerbated adolescents’ depressive symptoms [22].

However, since 2021, COVID-19 is classified as a category B threat in China. Between then and now, there have been no related reports in Yunnan, southwest China. Therefore, this study aimed to evaluate the epidemic trends of depressive symptoms in school-aged adolescents in Yunnan, evaluate the psychological influences on adolescents in the COVID-19 pandemic, and develop effective strategies to prevent and control the development of depressive symptoms in adolescents in the COVID-19 era in Yunnan, southwest China. This longitudinal observational study collected data from before the COVID-19 pandemic to during the COVID-19 pandemic (2018 to 2022) for analysis.

## Methods

### Study Design and Data Source

This longitudinal observational study used data from 2018 to 2022 [23]. In brief, the study participants were randomly cluster sampled from 3 surveillance prefectures in Yunnan (namely, Kunming, Honghe, and Pu’er) in 2018. The surveillance prefectures were then expanded to cover all prefectures (16 prefectures) in Yunnan from 2019 to 2022 (including the surveillance points in Kunming, Honghe, and Pu’er in 2018). The sampling strategy aimed to ensure representation by including both urban and rural areas in each prefecture. Specifically, 1 urban city and 1 rural county were randomly sampled from each prefecture. In the selected urban city, 5 schools were randomly selected as surveillance schools. In the rural county, 4 schools were randomly selected as surveillance schools (including 1 junior boarding school and 1 senior boarding school). Surveillance schools remained consistent from 2018 to 2022. All participants aged 12 years to 18 years were randomly selected from surveillance schools. All participants or their parents or guardians provided written informed consent. There were 222,269 participants aged 12 years to 18 years included in this analysis: 6547 in 2018, 34,122 in 2019, 34,026 in 2020, 34,419 in 2021, and 113,155 in 2022. There were 105,075 boys and 117,194 girls.

### Study Tool

This study measured depressive symptoms using the Center for Epidemiological Studies Depression Scale (CES-D), developed by Radloff [24,25] from the National Institute of Mental Health. This scale comprises 20 items, with 16 items assessing negative affect and 4 items assessing positive affect of depressive symptoms. CES-D scores range from 0 to 60. The CES-D has good predictive accuracy when compared with the full 20-item version. In 2009, CES-D was verified as suitable for Chinese adolescents (Cronbach α=0.88) [26]. The CES-D focuses on evaluating the frequency of current depressive symptoms related to emotions or mood, which made it suitable for comparing survey results across different time points [27].

### Depressive Symptom Status

Adolescents who participated in the study were categorized into 4 groups based on their CES-D scores: no depressive symptoms, ≤16; mild depressive symptoms, 17 to 20; moderate depressive symptoms, 21 to 25; severe depressive symptoms, ≥26. A total score >16 was defined as the positive presence of depressive symptoms.

### Conceptual Framework

**Figure 1:**
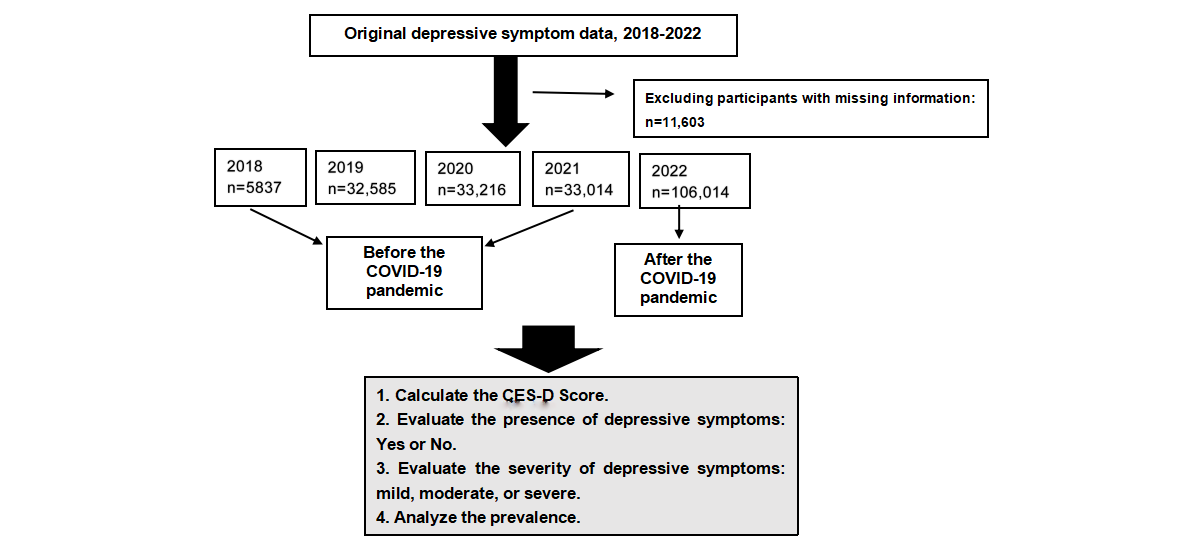
A conceptual framework of the trends of adolescents’ depressive symptoms in Yunnan, Southwest China, 2018-2022. CES-D: Center for Epidemiological Studies Depression Scale.

Figure 1 describes the conceptual framework of the study. This study collected continuous data per year from Yunnan adolescents from 2018 to 2022. This study defined 2018 and 2019 as before COVID-19, 2020 and 2021 as early COVID-19, and 2021 as the COVID-19 pandemic.

### Ethical Considerations

This study was approved by the Ethics Committee of Yunnan Centers for Disease Control and Prevention (approval number: 2021-05). Appropriate ethical considerations were followed during all the stages of this study. Participation in the study was entirely voluntary. All the students’ parents provided written informed consent for their children’s participation in this survey. The survey questionnaire and CES-D were distributed among respondents who provided their informed consent on the first page and clicked on the “agree” button after reading the purpose of the survey. The questionnaire and CES-D were anonymous to ensure the confidentiality and reliability of the data. Students or parents could refuse to participate in this survey. We analyzed data anonymously. The Ethics Committee approved this consent procedure. To ensure the data validity of the comparisons, the samples in each group were randomly selected in different survey years.

### Variables

The outcome variables in this study were average depressive symptom score and prevalence of depressive symptoms.

### Independent Variables

We selected age, gender, grade, residence, and ethnicity as the independent variables.

### Data Processing and Analysis

The field surveys and data collection were conducted by centrally trained professional and technical staff and executed to ensure comprehensive, reliable information gathering. There were significant differences in the distributions of participants by gender, age group, grade, ethnicity, and residence from 2018 to 2022. Therefore, these data were not comparable (*P*<.01). For comparability, the age-standardized and gender-standardized prevalences were calculated using the Chinese Census 2010 as a standard population. The age groups were set as 12 years to 13 years, 14 years to 15 years, and 16 years to 18 years.

The primary data analysis was conducted in 2023. We used frequencies, means, SDs, and percentages or proportions to describe adolescents’ depressive symptoms. Differences in the mean depressive symptoms by age, gender, and residence (urban and rural) were compared using ANOVAs. The division of urban and rural areas was based on the Chinese Administrative Division. The prevalence estimates for depressive symptoms in different survey years (2018, 2019, 2020, 2021, and 2022) were calculated by age, gender, ethnicity, and residence subgroups. Chi-square analysis was used to estimate *P* values for differences by gender, age, and residence subgroups, because high statistical power was achieved from the large sample sizes. Since depressive symptoms have normal, mild, moderate, and severe categories, we applied a rank sum test to fit the data. The association of COVID-19 with the risk of standardized depressive symptoms prevalence was identified using unconditional logistic regression analysis. All data were analyzed using SPSS 21.0 (IBM Corp) software. All the analyses included sample weights that accounted for the unequal probabilities of selection, oversampling, and nonresponse. Statistical significance was defined as *P*<.05.

## Results

### Population Demography

As shown in Table 1, the study sample was recruited from junior, senior, and vocational high schools in 32 counties of 16 prefectures in Yunnan, southwest China. A total of 222,269 adolescents aged 12 years to 18 years were included from 2018 to 2022, and 210,666 completed the physical examinations, resulting in a completion rate of 94.78%. All who completed the questionnaire and CES-D were included in the analysis (99,566 boys and 111,100 girls). The average age was 14.85 (SD 1.78) years.

**Table 1:** The demographic characteristics of adolescents aged 12 years to 18 years (N=210,666) in Yunnan, southwest China, 2018-2022.

Index		2018, n	2019, n	2020, n	2021, n	2022, n	*χ*^2^ (*df*)	*P* value
All		5837	32,585	33,216	33,014	106,014	—^a^	—
**Gender**							46.859 (1)	<.001
	Boys	2847	14,982	15,469	15,733	50,535		
	Girls	2990	17,603	17,747	17,281	55,479		
**Age** **(years)**							1092.734 (2)	<.001
	12-13	1572	8572	8602	9156	29,604		
	14-15	1828	10,756	10,944	10,795	40,251		
	16-18	2437	13,257	13,670	13,063	36,159		
**Grade**							6434.441 (2)	<.001
	Junior high school	2853	16,452	16,856	16,853	66,343		
	Senior high school	2149	12,550	12,794	12,429	36,420		
	Vocational high school	835	3583	3566	3732	3251		
**Ethnic** **ity**							53.473 (1)	<.001
	Han	3107	16,616	16,797	16,783	55,454		
	Minority	2730	15,969	16,419	16,231	50,560		
**Residence**							40,703.249 (1)	<.001
	Urban	3682	19,868	21,432	20,030	20,189		
	Rural	2155	12,717	11,784	12,984	85,825		

^a^Not applicable.

### Depressive Symptom Score Changes

**Figure 2:**
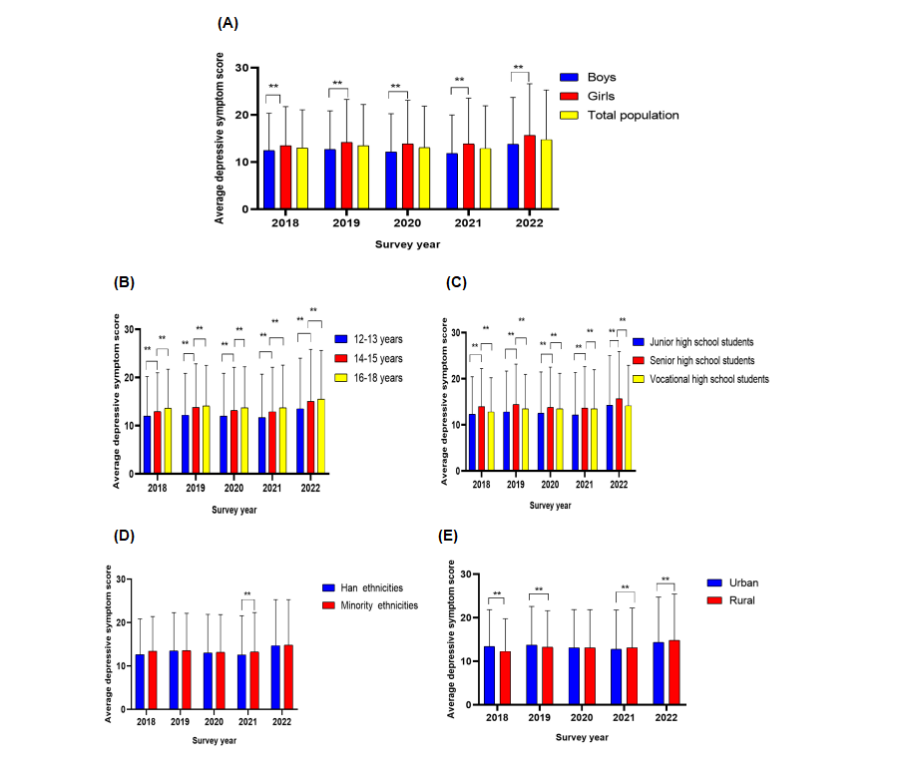
Changes in depressive symptom scores in different subgroups among adolescents aged 12 years to 18 years in Yunnan, southwest China, 2018-2022, by (A) gender, (B) age, (C) grade, (D) ethnicity, and (E) residence, as assessed using ANOVAs, except ethnicity.

As shown in Figure 2, the average depressive symptom score for the total sample increased from 13.00 (SD 8.12) in 2018 to 14.78 (SD 10.53) in 2022. The average increase was 0.36 points per year.

Regarding gender, the average depressive symptom score increased from 12.47 (SD 7.93) in 2018 to 13.79 (SD 9.95) in 2022 for boys. The average annual increase for boys was 0.26 points. The average depressive symptoms increased from 13.51 (SD 8.27) in 2018 to 15.68 (SD 10.95) in 2022 for girls. The average annual increase for girls was 0.43 points (*P*<.001).

For the different age groups, the average depressive symptom score increased with increasing age in any survey year (*P*<.001). The average depressive symptom score for those aged 12 years to 13 years increased from 12.04 (SD 8.18) in 2018 to 13.53 (SD 10.51) in 2022. The average annual increase was 0.30 points. The average depressive symptom score for those aged 14 years to 15 years increased from 12.97 (SD 8.03) in 2018 to 15.04 (SD 10.83) in 2022. The average annual increase was 0.41 points. The average depressive symptom score for those aged 16 years to 18 years increased from 13.64 (SD 8.10) in 2018 to 15.50 (SD 10.11) in 2022. The average annual increase was 0.37 points.

In the different grades, the average depressive symptom score decreased from senior high school to vocational high school to junior high school (*P*<.001). For senior high school students, the average depressive symptom score increased from 13.93 (SD 8.31) in 2018 to 15.70 (SD 10.22) in 2022. The average annual increase was 0.35 points. In vocational high school, the average depressive symptom score increased from 13.64 (SD 8.10) in 2018 to 15.50 (SD 10.11) in 2022. The average annual increase was 0.37 points. In junior high school, the average depressive symptom score increased from 12.35 (SD 8.12) in 2018 to 14.30 (SD 10.74) in 2022. The average annual increase was 0.39 points.

For different ethnicities, the average depressive symptom score was higher for minority ethnicities than for the Han ethnicity (*P*<.001). For those of Han ethnicity, the average depressive symptom score increased from 12.67 (SD 8.18) in 2018 to 14.72 (SD 10.58) in 2022. The average annual increase was 0.41 points. For minority ethnicities, the average depressive symptom score increased from 13.38 (SD 8.04) in 2018 to 14.83 (SD 10.47) in 2022. The average annual increase was 0.29 points.

For the different residence areas, rural adolescents had a higher average depressive symptom score than urban adolescents. For rural residents, the average depressive symptom score increased from 12.28 (SD 7.50) in 2018 to 14.87 (SD 10.56) in 2022. The average annual increase was 0.52 points. For urban residents, the average depressive symptom score increased from 13.42 (SD 8.44) in 2018 to 14.38 (SD 10.37) in 2022. The average annual increase was 0.19 points.

### Secular Trends in Depressive Symptom Prevalence

As Table 2 shows, the total crude prevalence of depressive symptoms was 33.55% (70,688/210,666). The crude prevalences were 29.03% (28,901/99,566) for boys, 37.61% (41,787/111,100) for girls, 27.66% (15,904/57,506) for those aged 12 years to 13 years, 34.47% (25,703/74,574) for those aged 14 years to 15 years, 37.01% (29,081/78,586) for those aged 16 years to 18 years, 30.99% (36,990/119,357) for junior high school students, 38.31% (29,250/76,342) for senior high school students, 29.72% (4448/14,967) for vocational high school students, 33.36% (36,285/108,757) for those of Han ethnicity, 33.76% (34,403/101,909) for minority ethnicities, 31.56% (26,893/85,201) for urban residents, and 34.91% (43,795/125,465) for rural residents.

After standardization, the standardized prevalence for all participants was 32.98%. The standardized prevalences of depressive symptoms were 30.49% before COVID-19, 29.29% in early COVID-19, and 36.33% during the COVID-19 pandemic. Per year, the standardized prevalences of depressive symptoms were 28.26% in 2018, 30.89% in 2019, 29.81% in 2020, 28.77% in 2021, and 36.33% in 2022. The standardized prevalences were 28.64% for boys, 36.87% for girls, 27.14% for those aged 12 years to 13 years, 33.99% for those aged 14 years to 15 years, 36.59% for those aged 16 years to 18 years, 34.94% for junior high school students, 34.58% for senior high school students, 34.58% for vocational high school students, 32.89% for those of Han ethnicity, 33.10% for minority ethnicities, 30.16% for urban residents, and 35.10% for rural residents.

**Figure 3:**
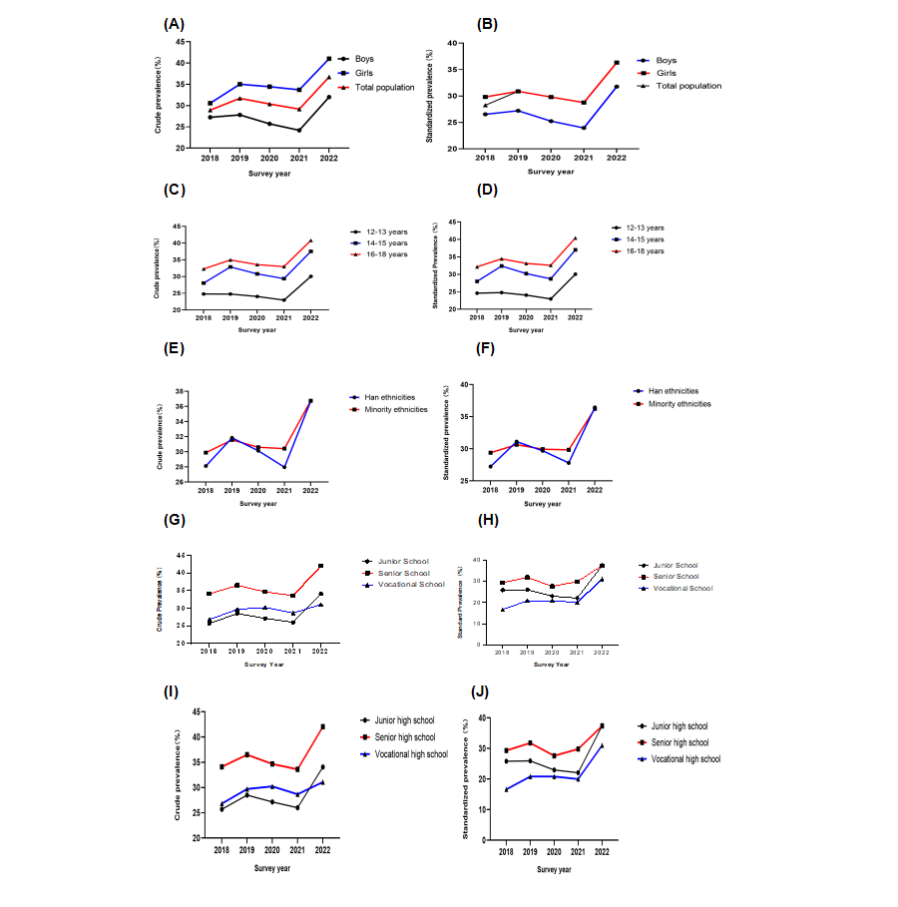
Secular trends in crude and standardized prevalences of depressive symptoms among adolescents aged 12 years to 18 years in Yunnan, southwest China, 2018-2022, by (A) and (B) gender, (C) and (D) age, (E) and (F) ethnicity, (G) and (H) grade, and (I) and (J) area of residence, as assessed using chi-square analyses.

As Figure 3 shows, the average annual incidence rates (AAIRs) of the standardized prevalences, in descending order, were 2.88% for vocational high school students, 2.32% for junior high school students, 2.31% for rural residents, 1.84% for Han adolescents, 1.8% for those aged 14 years to 15 years, 1.65% for those aged 16 years to 18 years, and 1.3% for girls.

As Figure 3 shows, comparing the standardized prevalences showed the following characteristics: (1) The rate of girls was higher than that of boys, and the acceleration rate was faster for girls than for boys; (2) with increasing age, the standardized prevalence increased, and the fastest acceleration rate was for adolescents aged 14 years to 15 years, followed by that for adolescents aged 16 years to 18 years; (3) there were no differences in prevalences between Han and minority populations, but the acceleration rate was faster for the Han population than for minority populations; (4) the rate for senior high school students was the highest, but the acceleration rate for vocational high school students was the fastest, followed by that of junior high school students; (5) the rate for rural residents was higher than that of urban residents, and the acceleration rate for rural residents was also quicker than that of urban residents.

**Table 2:** Crude prevalence of depressive symptoms among adolescents aged 12 years to 18 years in Yunnan, southwest China, 2018-2022.

Demographic characteristics		2018		2019		2020		2021		2022	
		Sample size, n	Depressive symptoms, n (%)	Sample size, n	Depressive symptoms, n (%)	Sample size, n	Depressive symptoms, n (%)	Sample size, n	Depressive symptoms, n (%)	Sample size, n	Depressive symptoms, n (%)
**Gender**											
	Boys	2847	776 (27.26)	14,982	4169 (27.83)	15,469	3977 (25.71)	15,733	3806 (24.19)	50,535	16,173 (32)
	Girls	2990	914 (30.57)	17,603	6167 (35.03)	17,747	6113 (34.45)	17,281	5826 (33.71)	55,479	22,767 (41.04)
**Age (years)**											
	12-13	1572	390 (24.81)	8572	2163 (25.23)	8602	2127 (24.73)	9156	2152 (23.50)	29,604	9072 (30.64)
	14-15	1828	513 (28.06)	10,756	3537 (32.88)	10,944	3372 (30.81)	10,795	3172 (29.38)	40,251	15,109 (37.54)
	16-18	2437	787 (32.29)	13,257	4636 (34.97)	13,670	4591 (33.58)	13,063	4308 (32.98)	36,159	14,759 (40.82)
**Grade**											
	Junior high school	2853	733 (25.69)	16,452	4688 (28.5)	16,856	4576 (27.15)	16,853	4383 (26.01)	66,343	22,610 (34.08)
	Senior high school	2149	733 (34.11)	12,550	4583 (36.52)	12,794	4436 (34.67)	12,429	4179 (33.62)	36,420	15,319 (42.06)
	Vocational high school	835	224 (26.83)	3583	1065 (29.72)	3566	1078 (30.23)	3732	1070 (28.67)	3251	1011 (31.10)
**Ethnicity**											
	Han	3107	874 (18.13)	16,616	5294 (31.86)	16,797	5064 (30.15)	16,783	4695 (27.97)	55,454	20,358 (36.71)
	Minority	2730	816 (29.89)	15,969	5042 (31.57)	16,419	5026 (30.61)	16,231	4937 (30.42)	50,560	18,582 (36.75)
**Residence**											
	Urban	3682	1149 (31.21)	19,868	6460 (32.51)	21,432	6481 (30.24)	20,030	5698 (28.45)	20,189	7105 (35.19)
	Rural	2155	541 (25.1)	12,717	3876 (30.48)	11,784	3609 (30.63)	12,984	3934 (30.30)	85,825	31,835 (37.09)
Total sample		5837	2847 (48.78)	32,585	10,336 (31.72)	33,216	10,090 (30.38)	33,014	9632 (29.18)	106,014	38,940 (36.73)

### Secular Trends in Different Categories of Depressive Symptoms

**Figure 4:**
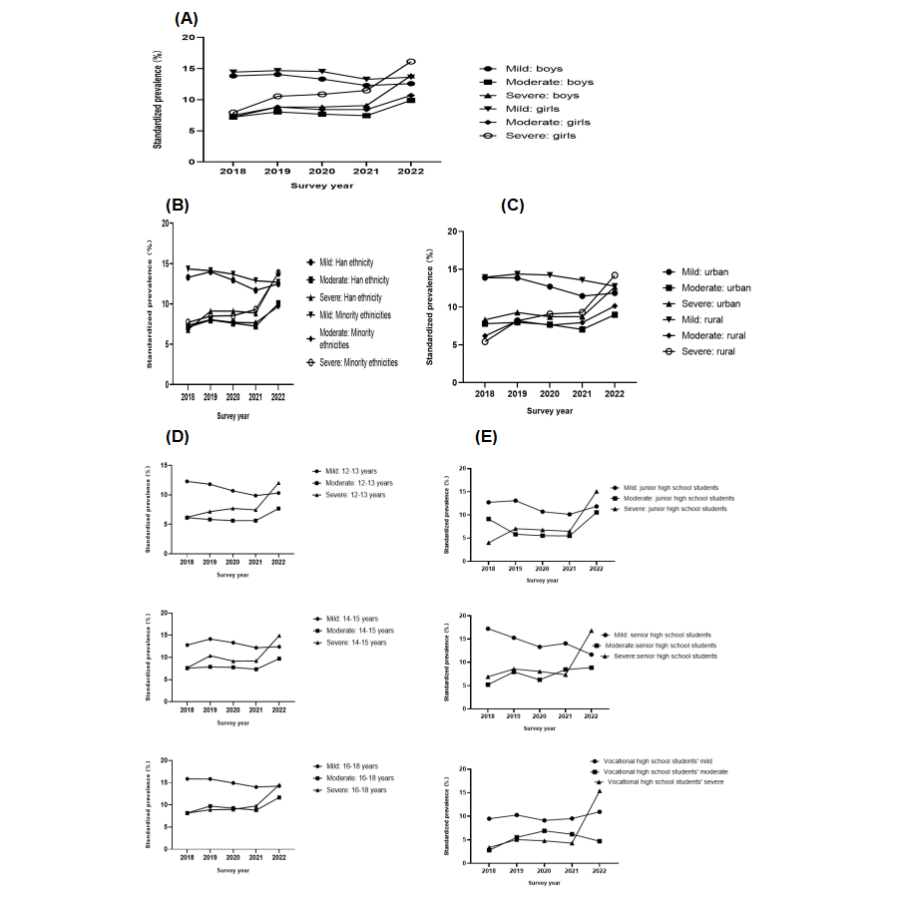
Secular trends in standardized prevalences of depressive symptom categories among adolescents aged 12 years to 18 years in Yunnan, southwest China, 2018-2022, by (A) gender, (B) ethnicity, (C) area of residence, (D) age of 12-13 years, (E) junior high school, (F) age of 14-15 years, (G) senior high school, (H) age of 16-18 years, and (I) vocational high school, as assessed using sum rank analyses.

As Figure 4 shows, the standardized prevalences of mild depressive symptoms decreased for all subgroups, and the rates of moderate and severe depressive symptoms both increased. Regardless of subgroup, severe depressive symptoms increased to a higher prevalence than moderate depressive symptoms. The rate of decrease in mild depressive symptoms was less than the rate of increase in severe depressive symptoms in the same subgroups.

The AAIRs of the standardized prevalences of severe depressive symptoms, in descending order, were 2.76% for rural residents, 2.4% for vocational high school students, 2.21% for junior high school students, 1.98% for senior high school students, 1.64% for girls, and 1.45% for those aged 14 years to 15 years.

### Relationship Between the COVID-19 Pandemic and Depressive Symptoms

**Figure 5:**
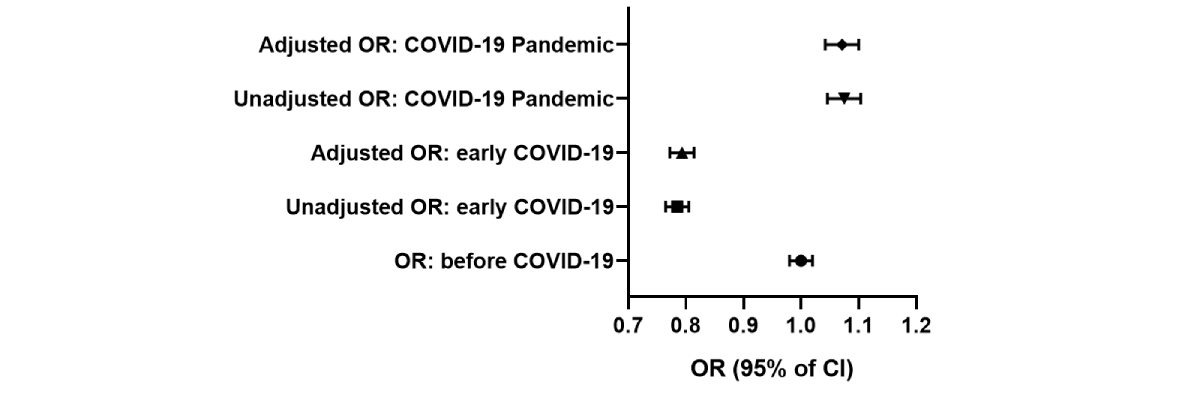
Risk for depressive symptoms based on COVID-19 stage among adolescents aged 12 years to 18 years in Yunnan, southwest China, 2018-2022, based on unadjusted odds ratio (OR) and OR adjusted for demographic characteristics (gender, age, grade, and ethnicity) and area of residence.

As Figure 5 shows, before adjustment, based on the odds ratio, the risk for depressive symptoms in early COVID-19 was 0.785 (95% CI 0.765-0.805; *P*<.001) that before COVID-19, and the risk of depressive symptoms during the COVID-19 pandemic was 1.074 (95% CI 1.046-1.104; *P*<.001) that before COVID-19.

In addition, after adjustment, the risk of depressive symptoms in early COVID-19 was 0.793 (95% CI 0.772-0.814; *P*<.001) that before COVID-19, and the risk of depressive symptoms during the COVID-19 pandemic was 1.071 (95% CI 1.042-1.100; *P*<.001) that before COVID-19.

Regardless of adjustment, the odds ratio was almost unchanged.

## Discussion

### Principal Findings

Yunnan Province is located in southwest China. It is China’s radiation center for south and southeast Asia as it is a border province that has one of the longest borders in China and borders Myanmar, Laos, and Vietnam. There are 25 border counties in 8 border prefectures.

The COVID-19 epidemic began in 2020 worldwide, and Yunnan experienced different degrees of impact. In the beginning, there was no epidemic in Yunnan, then Yunnan first reported COVID-19 patients in Ruili in 2021. There was no pandemic on a large scale in Yunnan before 2022. In 2022, the COVID-19 pandemic truly began in Yunnan. Previous studies found that the COVID-19 pandemic affected depressive symptoms in adolescents [28,29]. To date, there have been no relevant studies or reports in Yunnan Province. To evaluate the impact of the COVID-19 pandemic on the mental health of adolescents, reduce the effect or harm of psychological emergency syndrome, and promote the healthy, happy growth of adolescents, we conducted this study.

This study found that the standardized prevalence of depressive symptoms in all participants was 32.98% in Yunnan, southwest China. The standardized prevalences of depressive symptoms were 28.26% in 2018, 30.89% in 2019, 29.81% in 2020, 28.77% in 2021, and 36.33% in 2022. This finding was consistent with the 36.74% prevalence in Sichuan [30]. The rates in previous studies in China (14.5%, 2020) [31], Jiangsu (14.6%, 2022) [32], Korea (26.8%, 2021) [33], Finland (30.2%, 2021) [2], and the world (25.2%, 2021) [15] were lower than that in our study. The prevalence of depressive symptoms showed an increasing trend in severity. Moreover, this study showed that the standardized prevalence of mild depressive symptoms decreased, and the rates of moderate and severe depressive symptoms both increased. The AAIRs of the standardized prevalences of severe depressive symptoms, in descending order, were 2.76% for rural residents, 2.4% for vocational high school students, 2.21% for junior high school students, 1.98% for senior high school students, 1.64% for girls, and 1.45% for those aged 14 years to 15 years. Therefore, this study indicated that adolescents’ depressive symptoms represent an emergency public health problem in Yunnan, and the epidemic remains at a relatively high level compared with that of adolescents in other countries. The problem of depressive symptoms in Yunnan adolescents, especially for girls aged 14 years to 15 years and adolescents living in rural areas, should be given more attention. We need to conduct continuous surveillance to understand the situation of the epidemic and influencing factors and enable timely warning of this public health problem.

In addition, the standardized prevalences of depressive symptoms were 30.49% before COVID-19, 29.29% in early COVID-19, and 36.3% during the COVID-19 pandemic. Although the prevalence of depressive symptoms for adolescents during the COVID-19 pandemic in Yunnan was not double that before COVID-19 or in early COVID-19 [15], the prevalence of adolescent depressive symptoms during the COVID-19 pandemic was higher than that before COVID-19 or in early COVID-19. This study found that the risks of depressive symptoms were 0.793 (95% CI 0.772-0.814) times higher in early COVID-19 than before COVID-19 and 1.071 (95% CI 1.042-1.100) times higher during the COVID-19 pandemic than before COVID-19 in Yunnan. The AAIR was 1.61%. This confirmed that the impact of COVID-19 on the prevalence of depressive symptoms in adolescents was particularly strong.

Furthermore, this study found the following epidemic characteristics of depressive symptoms in Yunnan adolescents during COVID-19. First, the prevalence of depressive symptoms in girls (36.87%) was higher than that in boys (28.64%). The acceleration rate for girls was also faster than that for boys. Second, the prevalences were 27.14% for those aged 12 years to 13 years, 33.99% for those aged 14 years to 15 years, and 36.59% for those aged 16 years to 18 years. This shows that, with increasing age, the standardized prevalence increased. The acceleration rate was the fastest in adolescents aged 14 years to 15 years (1.80%), followed by adolescents aged 16 years to 18 years (1.65%). Third, there were no differences in prevalence between Han (32.89%) and minority (33.10%) populations. However, the acceleration rate for the Han population was faster than that for the minority population. Fourth, the rate for senior high school students was the highest (34.94%). However, the acceleration rate for vocational high school students was the fastest (34.58%), followed by that for junior high school students (34.58%). Finally, the rate for rural residents (35.10%) was higher than that for urban residents (30.16%). The acceleration rate for rural residents was also faster than that for urban residents. According to these findings, in Yunnan, rural areas should be key intervention areas. Vocational high schools should be emphasized as schools in which to implement interventions. Girls and all adolescents aged 14 years to 15 years should be the priority intervention populations.

According to previous studies, depressive symptoms are highly associated with internet addiction, depression disorders, self-injury, suicide idealization, and even suicide [34,35]. The key guiding principle is that we should implement psychological and lifestyle interventions as soon as possible [36]. Positive family communication, healthy dietary habits, regular physical activity [37-39], and life skills (eg, health-related knowledge, problem-solving, and emotion management [40,41]) are crucial for reducing depression and promoting healthy development in adolescents. Therefore, we must conduct joint interventions for adolescents with their families, communities, and schools. We should prepare to offer more mental health and other stress management services during public health emergencies [10]. For example, these services could strengthen family support to help parents understand emotional health issues in adolescents, providing family therapy; develop early interventions; and enhance social workers’ ability to provide virtual and phone-based assistance to adolescents. Schools could receive suggestions to find creative ways to encourage physical activity and allow students to enjoy music to reduce stress, anxiety, and loneliness [8].

This study also found that we need to continue monitoring to consistently observe the immediate and long-term effects caused by adolescents’ depressive symptoms. At the same time, it is important to observe the impact of timely psychological support and intervention on the future mental health of adolescents and evaluate the effect of different psychological intervention styles in future research.

### Limitations

This study has a few limitations. First, the data originated from a survey that involved self-reported CES-D scores for depressive symptoms, which may not align with an objective assessment by mental health professionals, and was conducted during an epidemic. Nevertheless, psychological impact and depression are based on personal feelings, and self-reporting was paramount during the COVID-19 pandemic. Second, when we designed this study, we only knew a little about COVID-19. Therefore, we did not measure neurological symptoms (eg, loss of smell or taste) that were discovered later to be associated with depressive mood. Last, because of the anonymous nature of the survey, we could not pair responses from the same individuals at the 5 time intervals.

### Conclusions

In summary, this study was the first to use a longitudinal, observational survey with successive follow-up data with adolescents aged 12 years to 18 years in Yunnan, southwest China. This study compared the impact of depressive symptoms from before COVID-19 through the COVID-19 pandemic. The standardized prevalences of depressive symptoms were 30.49% before COVID-19, 29.29% in early COVID-19, and 36.33% during the COVID-19 pandemic. In Yunnan, the risk of depressive symptoms in early COVID-19 was 0.793 (95% CI 0.772-0.814) that before COVID-19, and during the COVID-19 pandemic, it was 1.071 (95% CI 1.042-1.100) that before COVID-19. The AAIR was 1.61%. This revealed that the impact of COVID-19 on adolescents’ depressive symptoms was particularly strong. In the COVID-19 era, we should conduct continuous surveillance to understand the epidemic situation and influencing factors and enable a timely warning of adolescents’ depressive symptoms. In the meantime, we should also design efficient, joint interventions to decrease the prevalence of adolescents’ depressive symptoms.

## Abbreviations

AAIR: average annual incidence rate
CES-D: Center for Epidemiological Studies Depression Scale

## Data Availability

The raw data sets from this study can be available by emailing the corresponding author on reasonable request.
